# Dignity Therapy in Pediatrics: A Case Series

**DOI:** 10.1089/pmr.2020.0015

**Published:** 2020-08-06

**Authors:** Taryn Schuelke, Jared Rubenstein

**Affiliations:** ^1^ Palliative Care, Texas Children's Hospital, Houston, Texas, USA.; ^2^ Baylor College of Medicine, Houston, Texas, USA.

**Keywords:** dignity therapy, legacy, pediatric palliative care

## Abstract

***Objective:*** To report our first case series of Dignity Therapy modified for a pediatric palliative care population.

***Background:*** Dignity Therapy has been utilized successfully with terminally ill adult patients to help restore a sense of dignity and personhood as well as cope with existential distress near the end of life. To our knowledge, there are no published reports of this treatment modality in pediatric patients.

***Methods:*** The authors report the experience of a single-center case series of Dignity Therapy in a pediatric palliative care population. The adult Dignity Therapy process was adapted to fit the pediatric population and their families. Modifications are explained in some detail, and specific cases are shared to illustrate the process. The goal of this case series is to report on the application of Dignity Therapy to the pediatric population.

***Setting/subjects:*** Inclusion criteria for the cases series consisted of children and their families who were aware that death may occur soon, were English speaking, admitted to the hospital, and receiving care from the palliative care service.

***Results:*** Eight patients or their caregivers have completed Dignity Therapy thus far through our program. Four consented to publication of their experience. Three patients were adolescents and told their own story and the story of one younger nonverbal child was told by her family. All four participants reported that the intervention was acceptable and expressed gratitude for their final generativity document. No patient or family reported distress or negative effects from participation in Dignity Therapy.

***Conclusions:*** This case series describes how Dignity Therapy is possible with adaptations in the pediatric population, and how Dignity Therapy by proxy may be possible for caregivers of patients unable to tell their own story.

## Introduction

Chochinov et al. found that adults nearing the end of their lives experienced threats to intact personhood or lack of meaning.^1^ In response to these findings, Dignity Therapy was created as an intervention intended to support the following themes: (1) Generativity: the sense that the person has given something to future generations, (2) Continuity of Self: the sense that part of the person's legacy will continue, (3) Role Preservation: the sense that the person continues to play important roles in other people's lives, (4) Maintenance of Pride: the sense that the person can remain proud of their contribution to self and society, (5) Hopefulness: the sense that hope exists despite, or because of, their circumstances, (6) Aftermath Concerns: the concerns about what will happen to their family and personal responsibilities, and (7) Care Tenor: the quality of care provided to the person.^1^ Dignity Therapy has been shown to support adults in their final days with improvements in quality of life, sense of dignity, spiritual well-being and sadness.^2^ It has been applied to several cultures and cohorts for the past 15 years.^2–4^ However, to our knowledge there have been no published reports of Dignity Therapy in the pediatric population. In this case series, we describe our initial experience with adapting Dignity Therapy to the pediatric palliative care population.

## Methods

The authors attended the Dignity Therapy Training Workshop with Dr. Chochinov in Winnipeg, Canada. The workshop facilitators came from a variety of disciplines, including psychiatry, psychology, social work, and nursing. The workshop trainers stated that the intervention was designed to be delivered by any trained clinician, regardless of discipline. For our case series, the authors and providers of Dignity Therapy were a physician and a grief and bereavement specialist. The training provided three days of intensive skill building in Dignity Therapy, which consisted of literature review, instruction on each aspect of the intervention, editing practice, and observation of a live Dignity Therapy interview.

The process includes a recorded interview, editing session, and creation of a generativity document that the dying person provides to their chosen loved one(s). To participate in Dignity Therapy, a person must have come to the understanding that they are near the end of their life and be ready to engage in legacy work. Typically, patients and families who are discussing limitations of care, a hospice referral, or inquiring about legacy building opportunities meet inclusion criteria. For this case series, cognitively typical adolescents were included and were able to complete Dignity Therapy in its original form.

For younger preverbal or nonverbal children, a novel option of Dignity Therapy by proxy was used. Our model of Dignity Therapy by proxy was developed based on work being done by one of the Dignity Therapy Training Workshop facilitators using a caregiver-facilitated model for elderly patients with dementia (L. Montross, pers comm. about work not yet published, May 2017). If the child was unable to participate due to limitations of their diagnosis or their development (i.e., traumatic brain injury, nonverbal intellectual disability, or preverbal early childhood), the authors assessed for a proxy to complete the process. In these cases, as the patients were often nonverbal or preverbal, we empowered the caregiver to describe the full personhood of the child with detailed anecdotes about their life and personality as well as the shared life experience of the child and caregiver.

In a framing meeting, the dying child or their proxy learns about the Dignity Therapy process and chooses to whom the final generativity document will be provided. Examples are given of the types of questions that are asked during the interview to allow the dying child or proxy to think critically about what they would like to say before the interview (Table 1). The next step is the audio-recorded interview. The first half of the interview focuses on major life events and special memories. The second half of the interview explores any wisdom, wishes, or important words that still need to be said. Questions from the Dignity Therapy training are utilized in free-form conversation, and adapted to the specific child and family if necessary (Table 1).

**Table 1.** Standard Process for Dignity Therapy and Pediatric Adaptations

Adult process	Pediatric adaptation
**Setting:** Typically located in a hospice setting such as the dying person's home or an inpatient hospice facility.	**Setting:** Modified to be completed in the hospital setting such as at the patient's bedside or in a meeting room.
**Introduction:** Typically presented as some form of, “As you near the end of your life, there might be things you feel are left unsaid or memories that would like to pass on. Dignity Therapy is an opportunity to share your story and wisdom with your loved ones in the form of a printed document. Is that something that sounds interesting to you?”	**Introduction:** Modified as some form of, “You have a lot of great stories about your life. There is this thing called Dignity Therapy that you might like. You will get to tell your stories and share some hopes for your family that we can turn into a book. Does that sound like something you would like to do?”
**Framing meeting:** Typically, the adult shares who they want to receive the final document. The provider explains the process of Dignity Therapy further and gives some examples of questions that might be asked in the interview.	**Framing meeting:** Modified to be completed during the introduction if necessary, for timing. The child or proxy decides who they would like to receive the final document; this can include other children with similar diagnoses and prognoses.
**Interview questions:** Typically, the provider will ask standardized questions from the Dignity Therapy training, while still maintaining a feeling of casual conversation.	**Interview questions:** Modified to reflect the particular child's life experiences, or the proxy's perception.
For example	For example
1. “What are your hopes and dreams for your loved ones?”	1. “What is something you hope for your mom?” or “Is there something you dream your sister will do when she is older?”
2. “What are some of the important roles you played in your life?”	2. “What was it like being a brother?” or “What did you love about being a cousin?”
3. “Do you have any instructions you would want to pass along to others?”	3. “If you could make something special happen for your dad, what would that be?”
	For a proxy… “If your child could speak to you right now, what do you think they would say?”
**By proxy:** Typically, the proxy assists the dying adult with details of their story or reminds them of important moments.	**By proxy:** Modified to be provided entirely to the proxy of a preverbal child or child with nonverbal intellectual disability. In this case, the proxy could be a caregiver or older sibling. The proxy would share specific memories as they relate to the dying child and insights they gained from knowing that child. The intention is to share the dying child's story and experiences gained through caring for them.
**Final document:** Typically presented to the dying adult as a word only document.	**Final document:** Modified to include special art, pictures, or creative formatting that reflects the child's personality. If the child dies before the completion of the final document, it is then mailed or delivered to the chosen recipient.

If the child or proxy does not wish to answer a question, or it does not resonate with their life experiences, the provider moves on to additional questions. Once the interview is complete, the audio recording is transcribed by a transcription service and edited by the authors to create a narrative with consistent flow that sounds true to the voice of the interviewee. Once the draft is complete, it is read out loud to the interviewee. There are two goals in this meeting. First, the dying child or proxy is able to hear their document as it will be read by others. Second, it allows for any final editing that needs to be completed, that is, misspelled names or forgotten memories and sentiments. The final draft is formatted into the generativity document and printed.

Finally, the completed generativity document is provided to the dying child or proxy. Regarding time investment for the intervention, the initial interview and editing session take approximately an hour each. Interview transcription without a transcription service takes ∼8 hours of our own time and using a transcription service the turnaround time was 24 hours. Formatting and creation of the final generativity document takes approximately an hour. Altogether the process to create one final document can take two to four days so it was important to plan ahead and account for potential deterioration in participant's clinical status. Based on Dignity Therapy training guidelines, a contingency plan is discussed at the initial meeting in case participant dies or deteriorates before completion of the final document.

All participants are offered the option of having their story shared with others as part of their legacy. The authors offer the option to consent to have their stories shared with other members of their care team, with other patients, in presentations at academic meetings, and for publication in journals. For all stories shared in this article, interviewees or their family have provided consent and requested that their real names and stories be used as part of their legacy.

## Results

To date, the authors have completed eight total Dignity Therapy generativity documents for children ranging in age from 2 months to 22 years. Four were completed by proxy, and four were completed by the dying child or young adult.

Alex Unger was a 19-year-old boy who has end-stage heart disease. Alex wished to create his document for his parents and his younger sister. When asked what important roles he played in his life, he responded with a surprised, “I haven't ever thought of that before, but now that I'm thinking about it, I would say Protector.” Dignity Therapy gave Alex the opportunity to reflect on an aspect of life he had not thought about before. Alex was discharged home with hospice before completing the editing phase of the process, which posed a logistical problem for completing the document. The authors were able to arrange a video conference with him to complete his document. When hearing his story shared out loud, Alex remarked that it sounded “just like I was talking.” At the end of the editing session, Alex reflected on how his document “ will make everyone cry and laugh at the same time.” He also chose for his sister, who is an artist, to create the cover art. The final generativity document was sent in the mail.

Madeline “Maddie” Dibello was a nine-year-old girl with static encephalopathy, cerebral palsy, and nonverbal intellectual disability as a result of premature birth. The family expressed the desire to pursue Dignity Therapy before Maddie's death; however, she died before they could meet. They asked to return to the hospital to complete the interview in the week after her death. Maddie's family completed Dignity Therapy by proxy with all family members (mother, father, and three sisters) being interviewed together. The postmortem element of the interview created two unique opportunities in the Dignity Therapy process. First, it provided the family an opportunity to reflect on Maddie's life immediately after her death. The family spent the hour laughing and telling stories, which helped them feel “peaceful.” Second, the final document was much more reflective in nature than other documents. There were comments on how her death had already impacted the family. Another nonstandard element to Maddie's case was the full-family interview. Typically, only one person is interviewed. Transcribing and incorporating five voices into one coherent document were challenging. For the editing session, only the mother was present, and she commented on the “happy” nature of the document's final flow, which she felt reflected how the family felt about Maddie.

Miracle Akbar was a 16-year-old girl who was diagnosed with metastatic alveolar rhabdomyosarcoma. Before her death, Miracle began creating legacy items for her mother and six siblings, which made her an excellent candidate for Dignity Therapy. Miracle's disease affected her ability to speak, which made recording her interview difficult. The recording was successfully transcribed from a whisper. Her story contained significant hardship and tragedy, and the authors had concerns it may not be therapeutic for her to hear it retold. When Miracle's story was read back to her, she replied back with a firm, “ It's perfect.” Miracle wished for a party before she died. At that party, she read her completed Dignity Therapy document to everyone who attended and subsequently reported with a wry smile, “ Everyone cried. It was great.”

Shahd Shahroor is a 20-year-old girl with cystic fibrosis. When Shahd was 17, she had rapidly progressive respiratory failure. She acknowledged that her life may end soon and wanted to leave a strong legacy behind her. She loved telling stories, so Dignity Therapy was very much in line with her goals and personality. Her parents spoke Arabic, and her wish was for her story to be sent back to Palestine after she died. With that in mind, the document was translated to Arabic to achieve Shahd's wish. Remarkably, Shahd was able to receive a lung transplant. She is currently living at home with her family, enjoying her newfound time. She feels “ excited” hearing where her document has been shared for educational purposes.

After each interview, the provider asked for anecdotal experience of the process. Every child and proxy who completed the Dignity Therapy interview spoke of satisfaction with the process. Many felt it gave them a new perspective on the quality of life that had been lived. Several wished for their documents to be shared with other children and families who had a similar diagnosis. They all expressed gratitude for the opportunity after the final generativity document was completed. No children or families reported distress or negative outcomes from the process. The families who did not consent for their child's story to be shared felt it was “too personal” for others to read.

One interesting result the authors experienced with Dignity Therapy in pediatrics is a reticence of parents and clinicians to discuss impending death in a developmentally appropriate way with a child.^5,6^ When considering which children might meet inclusion criteria, the most common reason for exclusion was this issue.

## Discussion

The application of Dignity Therapy is possible with children. In the adolescent cases, the authors found it could be completed in its original design. For the preverbal or nonverbal pediatric patients, our experience suggests that the by-proxy model creates an opportunity for Dignity Therapy to be completed by a caregiver. Dignity Therapy may be an outlet for these families to share the depth of their child's life experience and can provide a time to reflect on their role as a caregiver. Involvement of siblings is also possible. In our experience, no child or caregiver who began the process stopped without completing it, which we believe suggests that this intervention can be done in the pediatric population.

We wanted to share some lessons learned over the course of compiling this case series. As believers in the Dignity Therapy model, we initially found ourselves trying as much as possible to adhere to the original protocol for interview and text-only generativity document creation. More and more, however, we found that adolescents and young adults requested modifications, specifically things such as adding important photos into the document as well as including their own artwork as cover art. In addition, for one of the by-proxy interviews, we experimented with an interview that involved a whole family, rather than a single caregiver. We feel that allowing these modifications ultimately enhanced the final documents and allowed further restoration of personhood.

There were also more practical lessons learned along the way. Initially, we were transcribing the interviews ourselves, which took significant time during an already busy workday. We were able to partner with our hospital's medical transcription service to have interviews transcribed and this saved us a significant amount of time. In addition, we began our interviews recording with a large professional-grade microphone. Over the course of the series, we transitioned to simply using our cell phones equipped with the secure transcription service to record. This was both less cumbersome and we feel also created a more organic feel for the interviewee.

We also wanted to briefly reflect on the children who were not eligible for Dignity Therapy because specifics of their diagnosis and prognosis were not discussed with them. Not only were these children limited on engaging in Dignity Therapy, but also advance care planning and memory making projects. This creates a barrier for legacy work in general and, in our opinion, is an area for improvement in our field.

## Conclusion

Dignity Therapy has shown benefit in the adult palliative care population and has been modified for use in multiple settings and cultures. We have found it possible to utilize Dignity Therapy in the pediatric population. All children and families that engaged in the process were able to complete it and expressed gratitude for the opportunity to create the Dignity Therapy document. Next steps are to refine the process of Dignity Therapy in pediatrics based on our experience and proceed with a feasibility study, with a goal to create a formalized program within our institution.

## Ethics

Given the unique nature of this study, consent was obtained through our hospital's media consent forms on an individual basis with each patient. Subsequent individual consents were obtained for each patient before journal submission and may be furnished upon request.
